# Hepatic Stellate Cells and Hepatocarcinogenesis

**DOI:** 10.3389/fcell.2020.00709

**Published:** 2020-08-05

**Authors:** Anna E. Barry, Rajkumar Baldeosingh, Ryan Lamm, Keyur Patel, Kai Zhang, Dana A. Dominguez, Kayla J. Kirton, Ashesh P. Shah, Hien Dang

**Affiliations:** ^1^Department of Surgery, Thomas Jefferson University, Philadelphia, PA, United States; ^2^Sidney Kimmel Cancer Center, Philadelphia, PA, United States; ^3^Department of General Surgery, UCSF East Bay, Oakland, CA, United States

**Keywords:** hepatic stellate cells, hepatocytes, hepatocellular carcinoma, tumor microenvironment, inflammation, fibrosis

## Abstract

Hepatic stellate cells (HSCs) are a significant component of the hepatocellular carcinoma (HCC) tumor microenvironment (TME). Activated HSCs transform into myofibroblast-like cells to promote fibrosis in response to liver injury or chronic inflammation, leading to cirrhosis and HCC. The hepatic TME is comprised of cellular components, including activated HSCs, tumor-associated macrophages, endothelial cells, immune cells, and non-cellular components, such as growth factors, proteolytic enzymes and their inhibitors, and other extracellular matrix (ECM) proteins. Interactions between HCC cells and their microenvironment have become topics under active investigation. These interactions within the hepatic TME have the potential to drive carcinogenesis and create challenges in generating effective therapies. Current studies reveal potential mechanisms through which activated HSCs drive hepatocarcinogenesis utilizing matricellular proteins and paracrine crosstalk within the TME. Since activated HSCs are primary secretors of ECM proteins during liver injury and inflammation, they help promote fibrogenesis, infiltrate the HCC stroma, and contribute to HCC development. In this review, we examine several recent studies revealing the roles of HSCs and their clinical implications in the development of fibrosis and cirrhosis within the hepatic TME.

## Introduction

Hepatocellular carcinoma (HCC) is the sixth leading cause of cancer related deaths in western countries (Choo et al., 2016; Bray et al., 2018), accounting for up to 90% of all primary liver cancers (Lozano et al., 2012). Although the percentage of HCC cases is dramatically higher in eastern Asia and most African countries, HCC is on the rise in the United States (U.S.) (Rawla et al., 2018). This trend is primarily due to increases in the incidences of chronic hepatic inflammation including fatty liver disease (FLD) (Nordenstedt et al., 2010) and chronic hepatitis C (HCV) infection (Kanwal et al., 2011). Although the distribution of HCC etiology varies between geographic regions, the most common etiology worldwide is viral hepatitis. In developing areas, such as Sub-Saharan Africa and Eastern Asia, hepatitis B virus (HBV) passed through vertical transmission at birth is the most common etiology (El-Serag, 2012). In developed worlds such as North America, HCV infection acquired later in life is endemic (Armstrong et al., 2006; Barazani et al., 2007; Wasley et al., 2008). In addition to viral hepatitis and FLD, alcoholic cirrhosis is a major contributing etiology of HCC; all of which can induce fibrosis, cirrhosis, and ultimately lead to development of HCC. This pathway to malignancy, driven largely through fibrosis, is supported by the fact that 90% of HCC patients have cirrhosis (Parkin et al., 2005; Ananthakrishnan et al., 2006).

**FIGURE 1:**
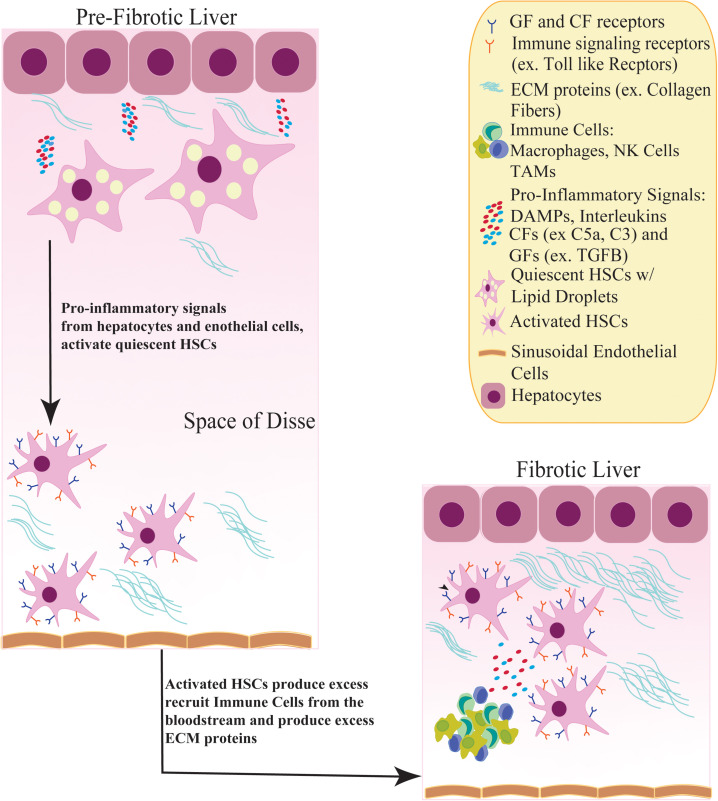
Activation of quiescent HSCs contribute to a fibrotic liver. In a pre-fibrotic liver, quiescent HSCs in the Space of Disse receive inflammatory signals, such as Damaged Associated Molecular Patterns (DAMPs) and Complimentary factors (CFs), from nearby hepatocytes and sinusoidal endothelial cells. In response, HSCs become activated, adopting a myofibroblast phenotype and losing its lipid droplets containing Vitamin A. Activated HSCs induce growth factor receptor signaling (TGFB, PDGF) and recruit immune cells such as NK cells and tumor associated macrophages (TAMs) from the bloodstream which produce more inflammatory stimuli. These inflammatory signals stimulate HSCs to secrete excess ECM proteins (ex. Collagen fibers), creating a fibrotic environment.

One of the main components in the development of fibrosis, cirrhosis and HCC (Wynn, 2008; Sokolovic et al., 2010) are the liver-specific pericytes, known as hepatic stellate cells (HSCs), which are located in the perisinusoidal space of the liver (Tsuchida and Friedman, 2017). Under normal conditions, HSCs exist in a quiescent state containing abundant lipid droplets of vitamin A (Blaner et al., 2009), and are highly sensitive to extracellular signals from fibrotic stimuli (Yin et al., 2013) including hepatitis, inflammation, or tissue injury (Wynn, 2008). In the presence of liver injury, HSCs are activated, transitioning from a quiescent to a myofibroblast phenotype with proliferative, migratory and invasive capabilities (Coulouarn et al., 2012; Carloni et al., 2014; Novikova et al., 2017). The well-known sequence of HSC activation can be split into two pathways. The first includes ‘initiation,’ which describes changes in HSC gene expression and phenotype rendering them more sensitive to paracrine stimuli. The second pathway includes ‘perpetuation,’ which amplifies the HSC-initiated phenotype via enhanced proliferation and proinflammatory signaling (i.e., damaged associated molecular patterns, DAMPs), interleukins, complimentary and growth factors from nearby damaged hepatocytes, endothelial cells and immune cells, resulting in promotion of fibrogenesis (Friedman, 2000) (Figure 1). Extracellular matrix (ECM) molecules (ex, type I and III collagen) are secreted by activated HSCs and accumulate to form scar tissue in the space of Disse (Friedman, 2008a) (Figure 1). This scar tissue functions to protect the liver from further damage; however, sustained activation of HSCs leads to chronic fibrosis and cirrhosis (Lee and Friedman, 2011; Friedman et al., 2013).

Activated HSCs play an essential role in fibrosis and hepatocarcinogenesis (Tsuchida and Friedman, 2017). Mediators in the activation of HSCs and the hepatic tumor microenvironment (TME) consist of transforming growth factor beta (TGFB), platelet derived growth factor (PDGF), connective tissue growth factor (CCN2, previously CTGF), vascular endothelial growth factor (VEGF), viral infection, focal adhesion kinase-matrix metalloproteinase 9 (FAK-MMP9) signaling, p53/21, phosphatidylinositol 3-kinase/protein kinase B (AKT) (PI3K/AKT), mitogen-activated protein kinase/extracellular signal-regulated kinase (MAPK/ERK), and interleukin 6/signal transducer and activator of transcription 3 (IL-6/STAT3) signaling pathways (Han et al., 2014; Fabregat et al., 2016; Makino et al., 2018). Furthermore, previous studies have shown HSC activation to be regulated by the tumor suppressor phosphatase and tensin homolog deleted on chromosome 10 (PTEN) (Takashima et al., 2009; Bian et al., 2012). These characteristics of activated HSCs and the role of the TME lay the foundation for exploring the relationship between the hepatic TME, activated HSCs, and hepatocarcinogenesis. This review will focus on the mechanisms regulating HSC activation and how they contribute to fibrosis, cirrhosis and HCC development (Wright et al., 2014; Coll et al., 2015; Das et al., 2020) and promising clinical therapies associated with HSCs (Dong et al., 2018; Li et al., 2020).

## The Role of Activated Hepatic Stellate Cells in Hepatic Fibrosis, Cirrhosis, and Progression to HCC

Hepatic fibrosis is a major risk factor for HCC development. Furthermore, activation of HSCs is a driver of hepatic fibrosis, cirrhosis and HCC (Tsuchida and Friedman, 2017). Due to the lack of effective liver fibrosis treatments, there is a need to better understand the molecular pathways and mediators of HSC activation to develop advantageous, targeted therapies for liver disease. Hepatic fibrosis is a result of chronic inflammation (post liver injury) characterized by secretion of excess ECM components, resulting in a wound healing response that will produce a “scar” in the liver (Friedman, 2008b; Lee and Friedman, 2011). Chronic inflammation can be induced by HBV/HCV infection, alcohol abuse, FLD (including non-alcoholic fatty liver disease and non-alcoholic steatohepatitis) or other metabolic disorders of the normal liver (Higashi et al., 2017). During post chronic liver inflammation, HSC activation supports the development of fibrosis and later cirrhosis, characterized by liver function impairment, portal vein hypertension and jaundice (Gines et al., 1987; de Franchis, 2000; D’Amico et al., 2006). Therefore, preventing the activation and proliferation of HSCs in cases of hepatic fibrosis has the potential to delay progression to HCC.

### The Role of DNA Methylation in HSC Activation

Recently, studies have begun focusing on the epigenetic regulation of HSCs to uncover the complexity of HSC activation (Tsuchida and Friedman, 2017). The tumor suppressor gene PTEN is an important negative regulator of HSC activation, which is silenced through promoter hypermethylation in tumors (Cairns et al., 1997; Salvesen et al., 2001; Soria et al., 2002; Furuta et al., 2004; Roman-Gomez et al., 2004; Mirmohammadsadegh et al., 2006; Wiencke et al., 2007; Tao et al., 2011; van Eggermond et al., 2011; Bian et al., 2012). Furthermore, the inhibition of PTEN activity leads to a constitutive activation of HSCs, which can perpetuate hepatic fibrosis (Bian et al., 2012). In addition, activated HSCs have been shown to possess altered DNA methylation and hydroxymethylation marks. For example, Page et al. (2016) showed that activated HSCs lose their fibrogenic phenotype when DNA methyltransferase 3a (DNMT3a) is suppressed in a carbon tetra chloride (CCl_4_) rat model of liver fibrosis *in vivo*. The continued discovery of novel mediators of HSC activation, including epigenetic regulators, will help better understand the role of activated HSCs in hepatic fibrosis.

### Pro-fibrogenic Cytokines in HSC Activation During Hepatic Fibrosis

Transforming growth factor beta is one of the key fibrogenic cytokines that drive hepatic fibrosis and regulate HSC activation (Fabregat et al., 2016; Tsuchida and Friedman, 2017; Dewidar et al., 2019). The bona-fide TGFB signaling pathway requires latent TGFB to be cleaved and activated by thrombospondin 1 (TSP1). Activated TGFB then binds to TGFB receptor 2 (TGFBR2), inducing phosphorylation of mothers against decapentaplegic homolog 2 and 3 (SMAD2, SMAD3) which translocate into the nucleus to regulate downstream gene expression of pro-fibrotic genes (Breitkopf et al., 2006; Friedman, 2008a; Liu et al., 2013; Meng et al., 2016; Tsuchida and Friedman, 2017; Murphy-Ullrich and Suto, 2018). TGFB can also activate the MAPK p38, ERK, and c-jun N-terminal kinase (JNK) pathways to regulate HSC activation (Engel et al., 1999; Hanafusa et al., 1999). Central to liver injury and HSC activation is a pathological increase in expression and activation of TGFB in the ECM (Tsuchida and Friedman, 2017). During HSC activation, TGFB targets and binds to HSCs, inducing phosphorylation of SMAD3 to promote production of type I and II collagen (Breitkopf et al., 2006; Friedman, 2008a). Activated HSCs also promote increased production of TGFB1 and TSP1 further enhancing the profibrogenic activities of TGFB (Breitkopf et al., 2005). Moreover, attenuation of TSP1 activated TGFB can be achieved with a TSP1 antagonist peptide and has been shown to decrease liver fibrosis in a dimethyl-nitrosamine liver fibrosis model (Li et al., 2017; Murphy-Ullrich and Suto, 2018), demonstrating the importance of this relationship. Along with TGFB, the DNA demethylase tet methylcytosine dioxygenase 3 (TET3) is also upregulated in mouse and human fibrotic livers (Xu et al., 2020). Xu et al. (2020) established TGFB1 stimulation could increase TET3 levels along with increased profibrotic gene expression in LX-2 cells, a human HSC cell line. Furthermore, TET3 was shown to upregulate the TGFB pathway genes TSP1 and TGFBR2, suggesting a positive feedback loop between TET3 and TGFB1 to promote HSC activation and hepatic fibrosis (Xu et al., 2020). In addition to TGFB signaling influencing HSC activation, hepatocyte produced extracellular matrix protein 1 (ECM1) has been shown to suppress TGFB levels and prevent HSC activation in an *in vivo* ECM1 knockout mouse model (Fan et al., 2019). Although TGFB remains a strong mediator of HSC activation and fibrogenesis, additional profibrogenic cytokines contribute to this process as well.

Another essential cytokine involved in HSC activation is PDGF (Tsuchida and Friedman, 2017). PDGF levels are increased in human cirrhotic livers compared to normal, healthy livers (Pinzani et al., 1996; Ikura et al., 1997; Stock et al., 2007; Tsuchida and Friedman, 2017). Upon liver injury in both humans and rodents, PDGF receptor beta (PDGFRB) expression increases in HSCs to drive HSC activation, proliferation and migration (Wong et al., 1994; Borkham-Kamphorst et al., 2004). Pdgf-C, a member of Pdgf family, is highly expressed on membrane receptors of hepatocytes in a transgenic Pdgf-c mouse model that resulted in dynamic liver fibrosis (Wright et al., 2014), suggesting that HSC activation may include Pdgf-c signaling. Pdgf is further supported as an effective activator of HSCs through the fibrotic role of Agrin (Agrn), a secreted proteoglycan induced by Pdgf-induced HSC activation in HCC in Diethyl nitrosamine (DEN)-induced HCC Sprague Dawley rat model (Lv et al., 2017). In this study the authors showed that Pdgf acts as an activator of the HSCs, which was inhibited by blocking the binding of Pdgf to its receptor. The authors also demonstrated that Agrin from activated HSC supernatant increased proliferation, metastasis, and invasion of SMMC-7721 (a human HCC cell line) and promoted epithelial to mesenchymal transition (EMT) (Lv et al., 2017). Overall, this study supports the role of PDGF-induced HSC activation resulting in fibrosis and HCC.

The matricellular protein CCN2, known for mediating fibrosis in various organs including the liver (Hall-Glenn and Lyons, 2011; Jun and Lau, 2011; Kodama et al., 2011; Lipson et al., 2012), has also been shown to activate HSCs and promote tumor progression via HSC secretion of the IL-6 and STAT3 *in vitro* (Makino et al., 2018). A potential clinical player in the cellular crosstalk between HCSs and HCC cells is stroma-derived fibroblast growth factor 9 (FGF9). Interestingly, a 2020 study conducted by Seitz et al. found that only activated HSCs expressed FGF9 compared to HCC cells. In HCC tissues, activated HSC overexpression of FGF9 reduced sensitivity to therapeutic agents and was associated with poor prognosis (Seitz et al., 2020), suggesting FGF9 as a potential therapeutic target and prognostic tool for HCC. Altogether, these findings support the notion of the growth factors PDGF-C and CCN2 as activators of HSCs and FGF9 as a potential clinical target for HCC.

### Association of Resident Liver Lymphocytes With Activated HSCs During Hepatic Fibrosis and Cirrhosis

Pathogenesis of liver fibrosis also involves resident liver lymphocytes including Type I and Type II Natural Killer T (NKT), Natural Killer (NK) cells and innate lymphoid cells (ILCs) (Wang and Zhang, 2019). Specifically, the interaction between activated HSCs and these hepatic lymphocytes is important (Wang and Zhang, 2019). The innate role of NKT cells is to defend against pathogens by recruiting circulating lymphocytes (Racanelli and Rehermann, 2006). Once activated, NKT cells can induce HSC activation via production of pro-inflammatory cytokines and release osteopontin and Hedgehog (Hh) ligands (Syn et al., 2012; Wehr et al., 2013) to aid in fibrosis development (Wang and Yin, 2015; Bandyopadhyay et al., 2016). However, it has been established that NK cells, along with NKT cells, protect the liver by preventing infection, tumor formation (Racanelli and Rehermann, 2006) and fibrogenesis (Melhem et al., 2006; Radaeva et al., 2006) in liver fibrosis mouse models (Muhanna et al., 2011; Gur et al., 2012; Hou et al., 2012) and HCV patients in various clinical studies (Glassner et al., 2012; Gur et al., 2012; Kramer et al., 2012). Furthermore, NK cells have an anti-fibrotic effect on HSCs by inducing activated HSC apoptosis (Radaeva et al., 2006). However, this is a temporary effect, and could result in apoptosis- resistant activated HSCs (Radaeva et al., 2007). Additionally, type 3 innate lymphoid cells (ILC3s) function as pro-fibrotic effectors in the liver (Muhanna et al., 2007; Bjorklund et al., 2016). Through co-culturing experiments with LX-2 cells, ILC3s promoted fibrogenesis via Interleukin-17A (IL-17A) and Interleukin-22 (IL-22), resulting in IL-22 inhibition of interferon gamma (IFNG) to indirectly enhance fibrogenesis (Wang et al., 2018). These data suggest an important role for resident liver immune cells in liver fibrosis through interactions with activated HSCs (Schon and Weiskirchen, 2014). Furthermore, they suggest the involvement of the innate immune system in relation to HSC activation in fibrosis; thus, warranting further study of the roles of activated HSCs in enhancing and suppressing fibrosis.

Even though stimulation of innate immunity has been shown to have a pivotal role in anti-viral and anti-tumor defenses in addition to fibrosis suppression, the regulation of innate immunity during chronic liver injury still needs to be elucidated. In a CCl_4_ mouse model, Infg induced NK cell activation was decreased in late liver fibrosis (advanced scarring) compared to early fibrosis (minimal scarring) (Jeong et al., 2011). The authors further demonstrated that the anti-fibrotic roles of NK cells are suppressed during advanced livery injury through increased expression of suppressor of cytokine signaling 1 (Socs1) and Tgfb (Jeong et al., 2011). This study also showed *in vitro* evidence of early activated HSC (4 days co-culture of HSCs and liver NK cells) induced NK cell activation via natural killer group 2 member D (NKG2D), whereas this was abolished in intermediately activated HSCs (8 days co culture of HSCs and liver NK cells) due to increased levels of TGFB1 and downregulation of NKG2D (Jeong et al., 2011). These results establish that although NK cells interact with activated HSCs to mitigate liver fibrosis during chronic liver injury, this process can be suppressed through increased TGFB and SOCS1 produced by activated HSCs *in vitro*.

In a 2017 study, Shi et al. (2017) showed that in liver cirrhosis patients, activated HSCs interact with purified NK cells through HSC-derived TGFB regulation of emperipolesis (the presence of an intact cell within the cytoplasm of another cell). This process was mediated through TGFB and evidenced by significantly reduced NK cell emperipolesis when activated HSCs were treated with an anti-TGFB antibody. The NK cells inside activated HSCs were also apoptotic as observed through positive terminal deoxynucleotidyl transferase dUTP nick end labeling (TUNEL) staining which indicates DNA fragmentation, a trait of cellular apoptosis (Kyrylkova et al., 2012). This suggests that activated HSCs reduce the anti-fibrotic roles of NK cells *in vitro* through activated HSC-derived TGFB and programmed death of NK cells to promote fibrosis (Shi et al., 2017). This study demonstrates that activated HSC-derived TGFB and NK cells work together to diminish NK cell anti-fibrotic capabilities and promote fibrosis in liver cirrhosis patients.

### Cellular Senescence of Activated HSCs During Hepatic Fibrosis

Senescence of activated HSCs has been shown to suppress liver fibrosis (Hoare et al., 2010; Jin et al., 2016; Nishizawa et al., 2016). In cancer, cellular senescence is a known tumor suppressive mechanism (Sager, 1991) activated by oncogenic stress (Campisi and d’Adda di Fagagna, 2007) and may also be essential for regulating liver fibrosis and cirrhosis (Tsuchida and Friedman, 2017). Cancer cell senescence is maintained by the tumor suppressor proteins p53/p21, p16^Ink4a^ and retinoblastoma (Rb) (Campisi and d’Adda di Fagagna, 2007; Collado et al., 2007). Furthermore, transgenic p53^–/–^ mice, with p53 knocked out specifically in HSCs, that were treated with CCl_4_ showed increased fibrosis compared to WT p53 mice. This phenotype indicates that activated HSCs have the ability to undergo senescence resulting in decreased liver fibrosis *in vivo* (Krizhanovsky et al., 2008). This study also revealed retained fibrotic lesions in p53^–/–^; Cdkn2a/Arf^–/–^ mice 20 days post CCl_4_ treatment, along with increased smooth muscle actin (Acta2), Tgfb, and Ki67 (marker of proliferative cells) expression. These findings suggest that activated HSCs during liver fibrosis can also evade cellular senescence to proliferate and secrete ECM components to begin developing the hepatic TME (Krizhanovsky et al., 2008).

HSC activation is one of the first responses to LSEC (liver sinusoidal endothelial cell) injury in the liver. LSECs serve as a permeable barrier between hepatocytes and the bloodstream and are characterized by fenestrations and a disorganized basement membrane, making them one of the most permeable types of endothelial cells (DeLeve and Maretti-Mira, 2017; Poisson et al., 2017). This permeability allows for efficient transport of solutes and metabolites throughout the liver. In addition, LSECs are an active contributor to the production of excess ECM proteins during liver fibrosis (Natarajan et al., 2017). Recent data shows that removal of senescent LSECs promotes liver fibrosis (Grosse et al., 2020). Through genetic lineage tracing mouse models, Grosse, et al. demonstrated that the majority of senescent cells were vascular endothelial cells, mostly LSECs in liver sinusoids, in addition to macrophages and adipocytes to a reduced extent. The authors also showed that both continuous and acute elimination of senescent cells disrupted blood-tissue barriers resulting in liver and perivascular tissue fibrosis. Overall, this study establishes that senescent cells involved in preventing liver fibrosis are primarily LSECs, rather than hepatic stellate cells (Grosse et al., 2020). While senescent LSECs serve as a barrier against fibrosis, the activation, proliferation and transformation of HSCs caused by liver injury, in addition to their ability to evade senescence once activated, are important developmental processes that provide a suitable microenvironment required for fibrosis, cirrhosis and later HCC development (Lashen et al., 2020).

## Activated Hepatic Stellate Cells Within the Hepatic Tumor Microenvironment

The interplay between liver tumor cells and the hepatic TME is crucial to the initiation and progression of HCC (Hernandez-Gea et al., 2013; Zhou et al., 2019). The TME is defined as a peritumoral space (Alfarouk et al., 2011; Joyce and Fearon, 2015; Spill et al., 2016) contributing to the acquisition of various hallmark traits of cancer, including sustained proliferative signaling and activation of invasion, metastasis, and angiogenesis (Hanahan and Weinberg, 2011). Furthermore, the TME can be divided into two major components: (1) cellular and (2) non-cellular. Activated HSCs are a part of the cellular component and exhibit essential biological functions such as promotion of fibrogenesis and ECM remodeling to positively influence HCC tumorigenesis (Friedman, 2008a; Amann et al., 2009; Coulouarn and Clement, 2014).

In addition to HSCs, cellular components of the hepatic TME include stromal hepatocytes, immune cells such as myeloid-derived suppressor cells (MDSCs) (Fu et al., 2007; Hoechst et al., 2008), tumor associated macrophages (TAMs) (Budhu and Wang, 2006; Budhu et al., 2006; Jeong et al., 2011), and cancer associated fibroblasts (CAFs) (Yin et al., 2019). Non-cellular components include cytokines such as Interleukin-6 (IL-6) (Budhu and Wang, 2006; Budhu et al., 2006) and Interleukin-22 (IL-22) (Jiang et al., 2011), growth factors such as VEGF (Coulouarn et al., 2012), TGFB (Thompson et al., 2015), PDGF (Tsuchida and Friedman, 2017), and CCN2 (Makino et al., 2018). Additional non-cellular components include matrix metalloproteinases (MMPs), their inhibitors (Novikova et al., 2017) and proteoglycans (Theocharis et al., 2010) (Table 1). The following studies will cover the different roles activated HSCs play in HCC progression through their interaction with the other cellular and non-cellular components of the hepatic TME.

**TABLE 1:** Components of the hepatic TME.

**Cellular**	**References**	**Non-cellular**	**References**
Stromal hepatocytes: connective tissue cells that provide support to the epithelial cells of the liver	Tahmasebi Birgani and Carloni, 2017	ECM Proteins (Matrix metalloproteinases, collagens, proteoglycans, lamins): key players in the invasive potential of HCC tumors by modulating the ECM	Novikova et al., 2017
Hepatic stellate cells: mesenchymal cells found in the liver that contribute to the hepatic TME by proliferating and promoting fibrosis when activated	Friedman, 2008a; Coulouarn and Clement, 2014	Growth factors (TGFB, PDGF, CCN2, VEGF, HGF): signaling proteins that stimulate the expression pathways of pro-fibrotic genes; stimulate HSC activation, proliferation and migration	Coulouarn et al., 2012; Thompson et al., 2015; Makino et al., 2018
Immune cells (Ex. Tumor Associated Macrophages and MDSCs): components of the hepatic TME that interact with activated HSCs by creating an immunosuppressed environment promoting HCC tumor growth and maintenance	Budhu and Wang, 2006; Budhu et al., 2006; Fu et al., 2007; Hoechst et al., 2008; Jeong et al., 2011	Cytokines (IL-6, IL-8, IL-22): small proteins involved in a range of cell signaling that help drive fibrosis, HSC activation, and contribute to angiogenesis	Chiu et al., 2014; Sevic et al., 2019
Cancer associated fibroblasts: type of cancer stromal cell critical to tumorigenesis regulation by possessing the ability to remodel the ECM and secrete proteins such as cytokines and VEGF	Yin et al., 2019		

*Both cellular and non-cellular components cooperate in the hepatic TME to aid in hepatocarcinogenesis.*

### Cellular Crosstalk Between Activated HSCs and Cellular Components of the Hepatic TME

The hepatic TME consists of various immune cells to create an immunosuppressed environment in order to maintain HCC tumor growth (Lu et al., 2019). Activated HSCs contribute to this immunosuppressed environment by secreting cytokines which induce MDSC expansion (Maher, 2001; Hui et al., 2004; Yu et al., 2004; Pan et al., 2008; Gabrilovich and Nagaraj, 2009; Hsieh et al., 2013). In an orthotopic liver tumor mouse model, activated HSCs significantly increased regulatory T cell (Treg) and MDSC expression to benefit HCC growth in the spleen, bone marrow, and tumor tissues (Zhao et al., 2014). Furthermore, activated HSCs secrete angiogenic growth factors to form new vasculature within the TME (Coulouarn et al., 2012; Heindryckx and Gerwins, 2015). These functions of activated HSCs create a link to the circulatory system for supplying nutrients to the tumor. Immune cells may also regulate activation of HSCs *in vitro*, demonstrated by Interleukin 20 (IL-20) activation of HSCs, resulting in upregulation of TGFB1 and type I collagen, and increased proliferation and migration of activated HSCs (Chiu et al., 2014). The same study further indicated that these fibrogenic phenotypes could be attenuated with an anti-IL-20 receptor (IL-20R1) monoclonal antibody, proposing IL-20 as a significant activator of HSCs and fibrogenesis. Taken together, activated HSCs may have an important role in promoting an immunosuppressed and angiogenic hepatic TME to support aggressive HCC cell growth.

### Crosstalk Between Activated HSCs and Non-cellular Components of the Hepatic TME

In addition to interacting with other hepatic TME cellular components, HSCs also respond to the non-cellular components of the liver TME (Hall-Glenn and Lyons, 2011; Jun and Lau, 2011; Kodama et al., 2011; Lipson et al., 2012). An example of such is the response to CCN2 produced from hepatic tumor cells (Makino et al., 2018). Makino et al. (2018) demonstrated that elevated CCN2 expression positively correlated with activated HSCs, indicated by smooth muscle actin (ACTA2) expression, in both mouse and human liver tumors. Furthermore, the authors showed that anti-CCN2 reduced IL-6 production in LX-2 cells and inhibited STAT3 activation in HepG2 (human HCC cell line) cells (Makino et al., 2018). This study was the first to establish HCC-cell-derived CCN2 activates HSCs in the TME, thus, accelerating the progression of HCC through cytokine production. These results also support the need for further exploration of CCN2 and other ECM proteins involved in the activation of HSCs.

While HSCs respond to growth factors such as CCN2; once activated, HSCs can also modulate the ECM through secretion and upregulation of proteins such as MMPs (Lachowski et al., 2019), which are needed for HCC tumor migration (Scheau et al., 2019). Studies have shown MMP2 and MMP9 (the most commonly studied MMPs in HCC EMT) to be important for the invasive potential of HCC tumors through degradation and remodeling of collagen in the ECM (Wang et al., 2014; Sun et al., 2018). Moreover, signaling between FAK and MMP9 is considered to be one of the main pathways that promotes HCC cell invasion and metastasis (Chen et al., 2010; Jia et al., 2011). Thus, it is plausible to speculate whether this signaling pathway is promoted through HSC activation. This hypothesis was explored by Han et al. (2014), who investigated whether activated HSCs promote FAK-MMP9 signaling *in vitro*. First, elevated numbers activated HSCs were shown to associate with tumor invasion of the portal vein, advanced tumor node metastasis staging, and lesser tumor differentiation. Thereafter, the number of activated HSCs, quantified by cytoplasmic ACTA2 expression, were positively correlated with the expression levels of phosphorylated FAK (p-FAK) and MMP9 in HCC. Furthermore, the authors used a co-culture experiment to demonstrate the activation of FAK-MMP9 signaling in HCC cells in the presence of activated HSC conditioned medium and with co-culture of activated HSCs. Additionally, inhibition of FAK-MMP9 signaling via small interfering RNA (siRNA) for FAK (siFAK) abrogated the migratory and invasive effects of activated HSCs on HCC cells (Han et al., 2014). These data show that FAK-MMP9 signaling is promoted by activated HSCS and plays a role in modulating metastasis of HCC following activation of HSCs; thus, highlighting the crosstalk between tumor cells and activated HSCs in the hepatic TME.

### Micro-RNA Involvement in HSC Activation in the Hepatic TME

In addition to ECM components such as MMPs and growth factors, recent advances have emphasized the significant roles played by micro-RNAs (miRNAs) in the TME (Cheng et al., 2015). This is demonstrated through the ability of miRNAs in tumor cells to transform the microenvironment by sustaining cancer hallmark traits and non-cell-autonomous signaling pathways (Suzuki et al., 2015). miRNAs are small non-coding RNAs (20-25 nucleotides in length) that regulate gene expression by binding to target mRNA transcripts through a seed sequence at the 5′ end of the miRNA (Bartel, 2004). In cancer cells, miRNAs are aberrantly expressed compared to normal cells, with expression patterns varying between different types of cancer cells (Karube et al., 2005; Lu et al., 2005; Merritt et al., 2008; Garzon et al., 2009). Additionally, activation and inactivation of HSCs can be controlled by profibrogenic and antifibrogenic miRNAs (Tsuchida and Friedman, 2017). Interestingly, miRNAs have been shown to possess dual roles as oncogenes and tumor suppressors in cancer cells (Zhang et al., 2007). The following studies investigate these dual roles in relation to the activation of HSCs in the hepatic TME.

Oncogenic miRNAs (oncomiRs) derived from the extracellular vesicles (EV) of HCC cells mediate communication between HCC cells and activated HSCs (Daugaard et al., 2017). Interestingly, crosstalk between miRNAs and TME components is partly mediated by exosomes, a type of EV produced in the endosome of eukaryotic cells that can transfer DNA, RNA and proteins to other cells (Zhang et al., 2015; Kosaka, 2016). Results from Li J. et al. (2019) found that EVs released by HepG2 and Huh7 (human HCC cell line) cells contained elevated oncomiRs. As a result, activated HSCs released their own EVs which stimulated HCC invasion, epithelial to mesenchymal transition (EMT) and activation of the AKT/ERK signaling pathway (Li J. et al., 2019). This suggests a positive feedback loop between exosomal oncomiRs of activated HSCs and HCC cells to promote hepatocarcinogenesis. Moreover, the authors observed upregulation of three specific oncomiRs: miR-21, miR-221, and miR-151. These results are further examples of the crosstalk between HCC cells and activated HSCs creating a pro-metastatic phenotype (Li J. et al., 2019).

Likewise, miR-1426 is an oncomiR shown to promote tumorigenesis, metastasis, and migration in multiple cancer types (Xu et al., 2019). Recently, a miRNA expression microarray study revealed a robust increase in miR-1246 in HCC cell lines when co-cultured with activated HSCs (Huang et al., 2020). These results reflect *in vitro* evidence that activated HSCs induce miR-1246 expression in HCC cell lines to promote metastasis. Moreover, miR-1246 and its target RAR related orphan receptor alpha (RORA), promoted EMT *in vitro* and *in vivo* in nude mice indicated by enhanced HCC cell migration, decreased *E*-cadherin, and increased vimentin protein expression. As a result of miR-1246 overexpression in PLC cells (human liver hepatoma cell line), the binding of RORA and beta-catenin (CTNNB1) in the cytoplasm was increased. This process was reversed with RORA knockdown, suggesting that the binding of RORA to beta-catenin prevents beta-catenin nuclear translocation and activation of the Wnt/beta-catenin signaling pathway. Furthermore, both miR-1246 and RORA were effectively used as independent prognostic markers in HCC tissue (Huang et al., 2020). This data suggests that miR-1246:RORA is a key component in the tumorigenic influence of activated HSCs on HCC cells, and that targeting the miR-1246:RORA axis may slow HCC progression (Huang et al., 2020).

However, miRNAs can also function as tumor suppressors in cancer cells (Zhang et al., 2007). For example, miRNA-212-3p has been shown to suppress cancer cell growth in other forms of cancer such as renal cell carcinoma (Gu et al., 2017) non-small-cell lung cancer (Tang et al., 2017) and glioblastoma (Tang et al., 2017). However, the effects, if any, in HCC remained unclear. Thus, Chen et al. (2019) examined the relationship between miRNA-212-3p and CCN2 in the hepatic TME. The authors showed that microRNA-212-3p inhibited proliferation of HCC cell lines through suppression of CCN2. Additionally, miR-212-3p was downregulated in HCC cell lines and tissues, and negatively correlated with vascular invasion and the absence of a fibrous tumor capsule. This fibrous capsule is formed by host liver mesenchymal cells, instead of HCC cells, and prevents possible invasion of HCC to the host liver (Ishizaki et al., 2001). These findings demonstrate a tumor suppressor role of miRNA-212-3p through its interaction with CCN2, a significant ECM component of the hepatic TME. Moreover, results from this study raise the question of whether microRNA-212-3p also inhibits the CCN2 mediated cytokine production in activated HSCs, given that the experiments from this study were carried out in HCC cell lines and tissues only.

### Activated HSC Regulation of Angiogenesis Within the Hepatic TME

The effects of activated HSCs on angiogenesis in HCC have also been investigated over the past decade. Angiogenesis within the TME is essential for tumor progression, metastasis and invasion (Mittal et al., 2014). Zhu et al. (2015) identified Interleukin-8 (IL-8) as a contributing factor to angiogenesis in HCC. Interestingly, IL-8 was highly expressed in HCC stroma and was mainly derived from activated HSCs rather than from HCC cells. Furthermore, an IL-8 neutralizing antibody was demonstrated to suppress tumor angiogenesis in Hep3B cells (a human HCC cell line) treated with conditioned media from activated HSCs. The authors also demonstrated similar results *in vivo* through a chick embryo chorioallantoic membrane (CAM) assay. Most recently, Lin et al. observed that activated HSCs are the primary source of secreted angiopoietin-1 (Ang-1) in human HCC cells *in vitro*. This not only describes the promotion of HCC angiogenesis through activated HSCs and Ang-1 expression, but also opens the potential of Ang-1 as an anti-angiogenic therapeutic target in HCC (Lin et al., 2020). These findings identify angiogenic factors produced by activated HSCs in the hepatic TME to promote hepatocarcinogenesis.

Intriguingly, a 2019 investigation found that activated HSCs induced angiogenesis in HCC via upregulation of glioma associated oncogene 1 (Gli-1), a member of the Hh signaling pathway (Yan et al., 2017). Furthermore, this study established that 3,5,4′-trihydroxy-*trans*-stilbene (trade name: Resveratrol), a polyphenol compound found in red-wine, grapes, berries and peanuts and believed to act as an antioxidant, also hindered HCC progression driven by HSCs through targeting Gli-1. Specifically, activated HSC induced angiogenesis in HCC via upregulation of Gli-1 expression, stimulated reactive oxygen species (ROS) production and increased HCC cell invasiveness. Resveratrol further abolished activated HSC-stimulated angiogenesis and suppressed ROS production and IL-6 and C-X-C chemokine receptor type 4 (CXCR4) expression in HepG2 cells by downregulating Gli-1 expression (Yan et al., 2017). In a separate study, Han et al. (2019) also described Resveratrol to possess tumor-suppressive effects through tumor microenvironment modulation across several types of cancers including HCC. This suggests the possibility of Gli-1 as a potential target for angiogenesis prevention in HCC.

## Quiescent Hepatic Stellate Cells

While activated HSCs play a major role in the formation of fibrosis and the hepatic TME, recent studies have also delved into the role of quiescent HSCs (qHSCs), as they are needed to maintain a healthy liver (Coll et al., 2015; Das et al., 2020). Uncovering the mechanisms preserving this phenotype could increase the scope of HSC targeted therapies.

Coll et al. (2015) conducted a study which examined these mechanisms and included a miRNA microarray analysis of isolated human qHSCs. The microarray revealed that HSCs express 259 miRNAs. In contrast, when HSCs were activated *in vitro*, 212 of these miRNAs were upregulated and the other 47 miRNAs were downregulated (Coll et al., 2015) suggesting a role for miRNAs in maintenance of qHSCs. Furthermore, the interactions between the miRNA target genes in qHSCs were also associated with HSC activation. Specifically, miRNA-192 was chosen for further *in vivo* study due to having 28 target genes in qHSCs and demonstrating decreased expression in cirrhotic liver samples compared to healthy samples. To elucidate the role of miRNA-192 in qHSCs *in vivo*, HSCs were isolated from two liver fibrosis mouse models and showed decreased miRNA-192 expression compared to healthy mouse HSCs. Furthermore, miRNA-192 overexpression resulted in inhibited Tgfb1 signaling and Pdgf-induced HSC migration in primary mouse HSC cells. This data supports miRNA-192 as a regulator of qHSCs through suppressing target genes needed for HSC activation (Coll et al., 2015). Thus, these findings support additional study into the miRNA-192 targeted genes involved in HSC activation to increase the scope of HSC targeted therapies.

Further supporting the significance of qHSCs, Das et al. demonstrated *in vitro* qHSC induction of cancer cell apoptosis via a caspase-independent mechanism (Das et al., 2020). This mechanism was established in rat hepatoma cells treated with qHSC conditioned media. The study determined that qHSC induction of apoptosis required increased apoptosis-inducing factor (AIF) expression, nuclear localization and DNA fragmentation, and resulted in eventual cell death (Das et al., 2020). This data illustrates the ability of qHSCs to increase toxicity and sensitivity to established chemotherapeutic agents, such as doxorubicin (Das et al., 2020), and could lead to augmentation of therapeutic strategies already in existence to increase their success.

## Clinical Implications and Relevance

Incidence of HCC in the U.S. has increased substantially in the past two decades (Singal et al., 2019; Das et al., 2020; Wu et al., 2020), and the American Cancer Society estimates 32,107 new cases of HCC will be diagnosed in 2020 along with 22,620 deaths (Society, 2020). Additionally, HCC incidence is three times higher in men than in women and the highest incidence is observed amongst patients greater than 70 years old with a steep mortality observed in patients ages 55–69 and older (Beal et al., 2017; Society, 2020). Unfortunately, over 80% of HCC patients present in advanced stages are not amenable to potentially curative surgical therapy, which combined with a paucity of effective systemic therapies, leads to a high morbidity and mortality rate (Zhou et al., 2018; Li D. et al., 2019). Ongoing investigation has focused on the process of tumor progression and potential therapeutic targets to create clinically relevant treatments for patients with HCC. Implicated in this research is the critical role of activated HSCs, from which 80–90% of HCC cells develop (Shiraha et al., 2020).

### Metformin

As mentioned previously, TGFB and PDGF induce HSC activation to contribute to the cellular crosstalk between tumor and stromal cells (Zhang and Friedman, 2012; Tsuchida and Friedman, 2017). GDF15, a member of the TGFB superfamily, is a biomarker for stress responses as a result of cancer treatment damage such as hypoxia and chemotherapy (Kelly et al., 2009; Corre et al., 2013) and may be a clinically relevant target for HCC. Common liver cancer therapy utilizes transarterial chemoembolization (TACE) that involves chemotherapy embolization-induced hypoxia to damage HCC cells; however, this therapy also induces stress in the surrounding HCC tissue (Dong et al., 2018). As a result of the intense cellular stress, GDF15 is secreted by the treatment-damaged HCC cells, and may have the ability to promote fibrosis through activated HSCs (Dong et al., 2018). To elucidate this process, Dong et al. (2018) demonstrated that HCC cells under “TACE-like” conditions showed increased levels of GDF15 via activation of the p38 MAPK, ERK1/2 and JNK pathways. Metformin, a common FDA approved drug, is known to target the JNK/p38MAPK pathway (Wu et al., 2011). Dong et al. (2018) further illustrated that in activated HSC cells, GDF15 promoted HSC proliferation and collagen production indicated by increased type I collagen protein levels cellular 5-Ethynyl-2′-deoxyuridine (Edu) incorporation, a thymidine analog which incorporates into the DNA of dividing cells (Salic and Mitchison, 2008). Conversely, Metformin was able to target the JNK pathway and suppress GDF15 expression resulting in decreased collagen synthesis and proliferation of activated HSCs *in vitro* and *in vivo* (Dong et al., 2018). These results suggest an opportunity to slow the progression of HCC by targeting activated HSCs with Metformin, a widely available drug.

### Sorafenib

Another FDA approved drug, Sorafenib, is a multi-target anti-angiogenic tyrosine kinase inhibitor (Hasskarl, 2014). Sorafenib was the first systemic therapy approved for treatment of HCC after it was shown to increase mean survival time by 2–3 months (Llovet et al., 2008; Cheng et al., 2009). A recently published study suggested that a novel system of biodegradable dendritic polymeric nanoparticles loaded with Sorafenib enhanced HCC therapy (Li et al., 2020). Through the MTT assay, this system induced higher cytotoxicity of HCC cells than a PEG-conjugated nanoparticle system containing Sorafenib and free-Sorafenib *in vitro*. In addition, tumor growth was significantly subdued in mice with HepG2 xenografts, with minimal side effects (Li et al., 2020). Moreover, a previous nanoparticle related study published in 2018 demonstrated that combined delivery of Sorafenib with a mitogen activated protein kinase (MEK) inhibitor using C-X-C motif chemokine receptor 4 (CXCR4)-targeted nanoparticles reduced hepatic fibrosis and prevented tumor development (Sung et al., 2018). This study further assessed the effects of Sorafenib on activated HSCs and established that combined delivery of Sorafenib and a MEK inhibitor through CXCR4-targeted nanoparticles prevented ERK activation in activated HSCs and had anti-fibrotic effects in the CCl_4_-mouse model (Sung et al., 2018).

### Bevacizumab

Continued interest in the critical role of activated HSCs in the regulation of angiogenesis will likely increase in light of recently released preliminary results of the IMbrave150 trial which challenges the longstanding paradigm of first line Sorafenib for advanced unresectable HCC. This study was a phase III randomized control trial of 336 patients who received either atezolizumab (PD-L1 inhibitor) plus Bevacizumab (VEGF-A inhibitor) or Sorafenib. Preliminary results from the trial demonstrated a significant overall survival benefit, with patients in the combination arm not reaching a median survival versus a median survival of 13.2 months with Sorafenib (*p* < 0.0001) (Cheng et al., 2019). Bevacizumab has been shown *in vivo* to decrease expression of profibrogenic genes TGFB and ACTA2, as well as decreasing overall HSC activation, altogether attenuating hepatic fibrosis in a CCl_4_-rat model (Huang et al., 2013). Although final results of the study have not yet been published, it is widely expected to be practice changing and highlights the importance of both the immune and angiogenic crosstalk in the hepatic TME and HCC progression.

### Potential Alternative Therapies

Finally, there is an interest in therapeutically targeting the cytokine-HSC interaction. An IL-8-neutralizing antibody (Zhu et al., 2015) and an anti-CCN2 neutralizing antibody which lead to reduced IL-6 production (Makino et al., 2018) have both shown therapeutic potential in suppressing tumor progression of HCC *in vitro* and *in vivo* with a xenograft murine model. These neutralizing antibodies, which both target interleukin cytokines, are potential clinical therapies focusing on the relationship between activated HSCs and TME. Additionally, a 2015 study established the angiogenin inhibitor neomycin as a potential HCC therapy. This study demonstrated that neomycin decreased HSC activation with conditioned media or recombinant angiogenin *in vitro* (Barcena et al., 2015). Furthermore, neomycin administration reduced tumor growth of HepG2-LX2 cells co-injected into mice, suggesting that angiogenin secretion by HCC cells favors tumor development via induction of HSC activation and ECM remodeling. These findings not only suggest that targeting angiogenin signaling may be of potential relevance in HCC management, but also establishes neomycin as a potential clinical treatment for HCC.

## Discussion

Hepatic stellate cells activation is the central event of hepatic fibrosis and the development of cirrhosis and HCC. A fundamental gap in knowledge is the crosstalk between activated HSCs, the hepatic ECM and HCC tumor cells. Recent studies have focused on targeted molecular therapeutic strategies for liver fibrosis and cirrhosis (van der Heide et al., 2019). Thus, utilizing multiple biomarkers may lead to optimized early detection of HCC (Ismail and Pinzani, 2011; Tuohetahuntila et al., 2017). Data published in 2017 suggested a new therapeutic option to target and increase NK activity in patients with chronic hepatitis infection preceding hepatic fibrosis (Shi et al., 2017). Other recent studies discussing potential targeted therapies such as PDGF-C and TGFB are under exploration. Ultimately, elucidating the mechanistic links between activated HSCs through all stages of fibrosis and cirrhosis will lead to a better understanding of HCC tumorigenesis.

Due to the intricate relationship between the hepatic TME, tumor development and HCC progression, recent studies have begun focusing on the role of activated HSCs, one of the prominent factors involved in the hepatic TME. The hepatic TME provides a niche which includes both cellular components, such as HSCs and non-cellular components, being the ECM and ECM proteins. As activated HSCs become ECM producing cells during liver fibrosis, secreted chemokines, cytokines, and growth factors prime the overall TME for supporting HCC proliferation.

**FIGURE 2:**
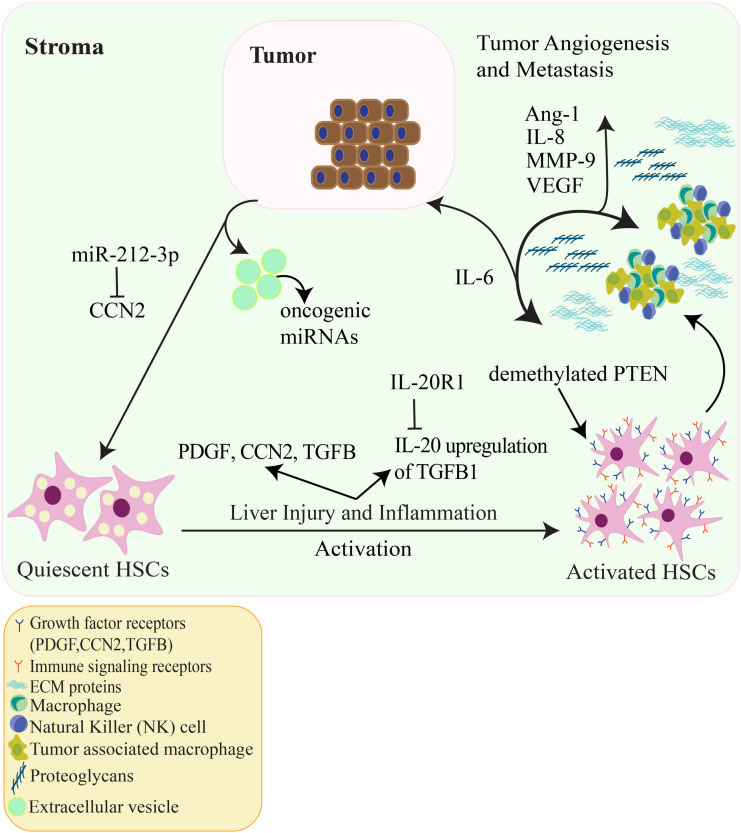
The relationship amongst HCC tumor cells, tumor angiogenesis and HSCs in the hepatic TME. Quiescent HSCs are activated by numerous factors such as liver damage, inflammation, PDGF and TGFB signaling, hepatitis and viral infection. Additionally, HSC activation can be epigenetically regulated by demethylation of the tumor suppressor PTEN and IL-20 activation of HSCs via upregulation of TGFB1. Activated HSCs go on to produce ECM proteins and alter the ECM accompanied by proteoglycans and VEGF. Cytokines such as IL-6 are released which promote HCC tumor cell proliferation. CCN2 produced from HCC cells can activate HSCs, in contrast, CCN2 is inhibited through the oncogenic miRNA (oncomir) miR-212-3p which decreases HCC cell invasion *in vitro*. Other oncomirs secreted by HCC extracellular vesicles can regulate signaling between HSCs and HCC cells. In addition, IL-8, MMP-9, and Ang-1 contribute to tumor-associated angiogenesis. These factors allow for HCC tumor proliferation, invasion, and metastasis.

Owing to the complexity of the TME, it is difficult to therapeutically target one pathway as they all have functional redundancies; however, targeting a pivotal cellular component of the hepatic TME, such as activated HSCs, may be more feasible. Pertinent to this review, studies focusing on the role of activated HSCs in the TME could lead to activated HSC targeted therapies that may affect activated HSC related factors such as TGFB, PDGF, MMP-9, CCN2, and oncogenic miRNAs. Other targets that warrant further study and serve as promising areas for therapeutic exploration include IL-8, Ang-1, and Gli-1. A proposed schematic illustrating the relationship between activated HSCs in the hepatic TME and HCC is shown in Figure 2.

Collectively the literature covered in this review outline the significance of activated HSCs in the hepatic TME. In addition to cellular crosstalk within the TME, activated HSCs play a crucial role in HCC progression through the TME’s non-cellular components. These recent studies provide examples of cytokines, growth factors, ECM components and microRNAs, which are all crucial non-cellular components of the hepatic TME involved in the relationship between activated HSCs and HCC development.

This review also concentrated on clinical implications that highlight potential therapies for HCC through targeting activated HSCs and their relationship with HCC cells. In addition to enhancing the efficacy of current therapeutic agents such as Metformin, Sorafenib, and Atezolizumab/Bevacizumab, potential alternative therapies include neomycin and neutralizing antibodies against IL-22 and CCN2.

A deeper understanding of how the hepatic TME, most notably activated HSCs, interacts with the primary tumor and non-tumor cells will propel advances in effective diagnostic and prognostic tools. Ongoing investigations are imperative in order to develop more effective treatments for HCC and augment current therapies to increase their success.

## Author Contributions

AB and RB equally drafted the review, compiled the authors’ contributions and references, wrote the manuscript, and produced the table and figures. RL, KP, KZ, DD, RB, AS, and KK contributed by writing and editing various sections within the manuscript. HD provided the overall support, intellectual input, critical evaluation, and all rounds of editing. All the authors read and approved the manuscript.

## Conflict of Interest

The authors declare that the research was conducted in the absence of any commercial or financial relationships that could be construed as a potential conflict of interest.
